# Editorial: Experimental models and model organisms in cardiac electrophysiology: opportunities and challenges

**DOI:** 10.3389/fphys.2023.1254596

**Published:** 2023-09-06

**Authors:** Bianca J. J. M. Brundel, Chris Jopling, Igor R. Efimov, Flavien Charpentier, Yoram Etzion

**Affiliations:** ^1^ Department of Physiology, Amsterdam Cardiovascular Sciences, Amsterdam University Medical Centers, VU Universiteit, Amsterdam, Netherlands; ^2^ Institute of Functional Genomics, University of Montpellier, CNRS, INSERM, LabEx ICST, Montpellier, France; ^3^ Department of Biomedical Engineering, Northwestern University, Chicago, IL, United States; ^4^ Department of Medicine, Northwestern University, Chicago, IL, United States; ^5^ Nantes Université, CNRS, INSERM, L’institut Du Thorax, Nantes, France; ^6^ Department of Physiology and Cell Biology, Ben-Gurion University of the Negev, Beer-Sheva, Israel

**Keywords:** experimental models, cardiac electrophysiology, arrhythmia, model organisms, cardiac electrophysiologic technique

Cardiac diseases are the main cause of death worldwide and their incidence and prevalence are likely to increase because of ageing of the population and unhealthy changes in lifestyle. Prevailing theories about the mechanisms of cardiac disease onset, encompass molecular and electrophysiological changes in the cardiac tissue and cells. Insights into the underlying molecular mechanisms are essential to create a deeper understanding of key concepts of cardiac disease development. Moreover, mechanistic insights may drive the development of novel technologies to explore further mechanisms as well as diagnostic and therapeutic tools for cardiac disease management in the clinic.

In this Research Topic “*Experimental Models and Model Organisms in Cardiac Electrophysiology: Opportunities and Challenges*” various experimental model systems are discussed that enable mechanistic studies on molecular mechanisms and electrophysiological changes that drive cardiac diseases. Experimental model systems include induced pluripotent stem cell (iPSC)-derived cardiomyocytes, as well as cultured neonatal monolayers of cardiomyocytes and fibroblasts. The contribution of Carvalho et al. describes the potential of iPSC-derived cardiomyocytes from healthy donors as a disease model to study electrical properties. By testing the action potential variability of 780 cardiomyocytes from six healthy heart donors, they observed that the action potential data per cell line, per differentiation protocol and per batch fluctuates. This has important consequences for mechanistic studies on arrhythmias. Further standardization of iPSC cardiomyocyte culture is needed for arrhythmia research. In line with Carvalho, the paper of Isamili et al. points out the biological and methodological issues researchers have to consider when working with iPSC-derived cardiomyocytes. Challenges include the variation in ion-channel currents, action potential durations, and atrial phenotype of atrial cardiomyocytes compared to isolated adult human cardiomyocytes. Despite these drawbacks, the authors are hopeful as they expect that the concerted expertise of experimental electrophysiologists and stem cell experts will eventually solve the challenges.

An innovative emerging model system in arrhythmia research is the use of (human) living myocardial tissue slices. Amesz et al. provide an overview of the potential of utilizing living myocardial slices in electrophysiological studies. The authors state that human atrial and ventricular tissue slices of 150–400 µm thickness (6–17 cardiomyocyte layers) can be used for action potential recordings, optical mapping, and extracellular field potential mapping. So far, living myocardial tissue slices hold the promise to facilitate detailed research on cardiac arrhythmias. Optogenetic light-based pacing is an emerging technique to manipulate cardiac activity in a spatial- and temporal-specific manner and improve our understanding of arrhythmogenic mechanisms. Marchal et al. show their results on the application of low-intensity, sub-threshold illumination to selectively manipulate cardiac electrical activity in defined areas of the heart. This approach enables the study of conduction slowing and repolarization heterogeneities during cardiac disease.

In addition to innovative model systems and technologies as described above, novel methodological insight into the interpretation of ECG data may improve the identification of cardiac arrhythmias. Mulla et al. review differences in rate-adaptation of ECG properties in mice and rats compared to humans. Although mice and rats have been used for decades as model systems to study cardiac arrhythmias, conflicting data on QT interval rate-dependence still exists, and therefore the authors state that the empirical ways by which QT intervals are corrected in rodent studies should be revisited. In addition to improved interpretation of ECG data in rodents, optimal programmed electrical stimulation is a prerequisite to assess atrial fibrillation susceptibility. Murphy et al. provide important insight into the optimal pacing protocol to elicit an atrial fibrillation phenotype in mice. Their main message is that for each study, an individualized protocol should be developed. Amorós-Figueras et al. describe a pig model for atrial arrhythmia by scarring of the atrium. Scarring is induced via selective occlusion of the atrial branches, resulting in atrial infarction in the left atrium, and low voltage of bipolar electrograms in affected areas. This model may have potential applicability for studying atrial arrhythmia mechanisms. According to Alameh et al. improved characterization of ion channel activity is required to more accurately study the role of hERG, the pore-forming subunit of the rapid component of the delayed rectifier K^+^ current, in ventricular repolarization. Mutations in hERG are associated with Long QT syndrome, but most identified variants have unknown or unclear functional consequences and are thus classified as variants of unknown significance. The authors advocate for a unique homogeneous protocol that could ultimately facilitate Long QT syndrome management. Lastly, Ernault et al. study the impact of atrial fibrosis on the development of atrial fibrillation. They describe that the selective disruption of primary cilia in fibroblasts via Ift88 knock-out induces extracellular matrix production, decreases conduction velocity, increases the number of block lines, and increases the risk or reentrant arrhythmias without changing the action potential (AP) characteristics of co-cultured cardiomyocytes with fibroblasts. The data suggest that dysregulation of primary cilia causes fibrosis and hinders myocardial conduction, thereby producing an arrhythmogenic substrate.

In summary, multiple model systems and technical approaches have been developed to study molecular and electrophysiological changes that drive cardiac arrhythmia in recent years. Novel mechanistic findings are important as they will fuel the identification of druggable and diagnostic targets, to ultimately improve clinical arrhythmia management. Hereto, a novel standardized methodology for the design of the models, induction of arrhythmias, and interpretation of the data is of prime importance. This Research Topic provides insights into all items relevant for solid translational research on cardiac arrhythmias.

